# Squamous Cell Carcinoma of Suprapubic Cystostomy Site in a Patient with Long-Term Suprapubic Urinary Catheter

**DOI:** 10.1155/2017/7940101

**Published:** 2017-11-12

**Authors:** Sasikumar Subramaniam, Gowribahan Thevarajah, Karunadasa Kolitha, Nandasena Namantha

**Affiliations:** ^1^Department of Urology, Colombo North Teaching Hospital, Ragama, Sri Lanka; ^2^Department of Plastic Surgery, Colombo North Teaching Hospital, Ragama, Sri Lanka

## Abstract

Patients with long-term suprapubic cystostomy can rarely develop squamous cell carcinoma (SCC) of the suprapubic cystostomy tract. In addition to the few reported cases in the literature, this paper reports a case of suprapubic cystostomy SCC in an 88-year-old man without bladder involvement. Vigilance about any abnormal lesion at the site of suprapubic cystostomy is important among health providers and patients for early detection of SCC.

## 1. Introduction

Squamous cell carcinoma contributes to 3% of bladder malignancies [1]. In patients with long-term suprapubic cystostomy, squamous cell carcinoma (SCC) can rarely arise from the suprapubic cystostomy tract itself and this has been reported only in a limited number of cases in the literature.

We report a case with suprapubic cystostomy tract SCC without involvement of the bladder and its management.

## 2. Case Report

An 88-year-old male patient presented to us with large fungating growth encircling the suprapubic cystostomy site of eight-month duration with gradual expansion in size. He underwent several surgical procedures for long urethral stricture, and as the measures finally failed, he has been on permanent suprapubic cystostomy for 25 years.

**Figure 1:**
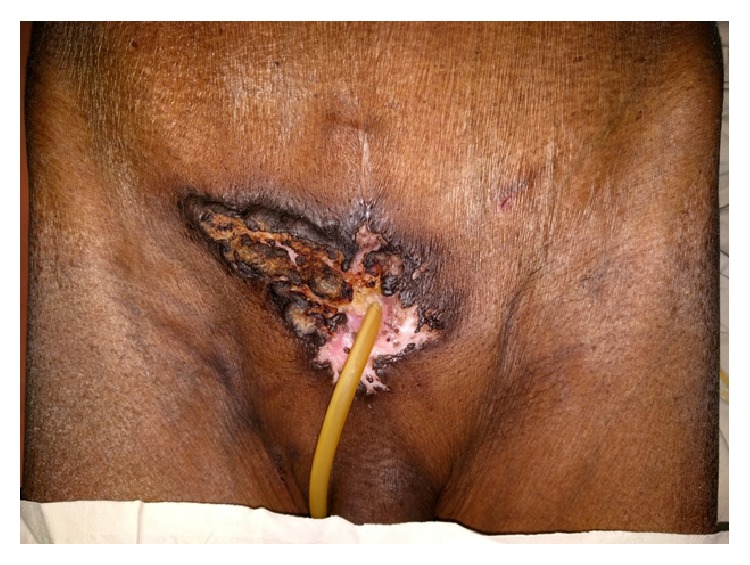
Suprapubic cystostomy site growth with urinary catheter in situ.

**Figure 2:**
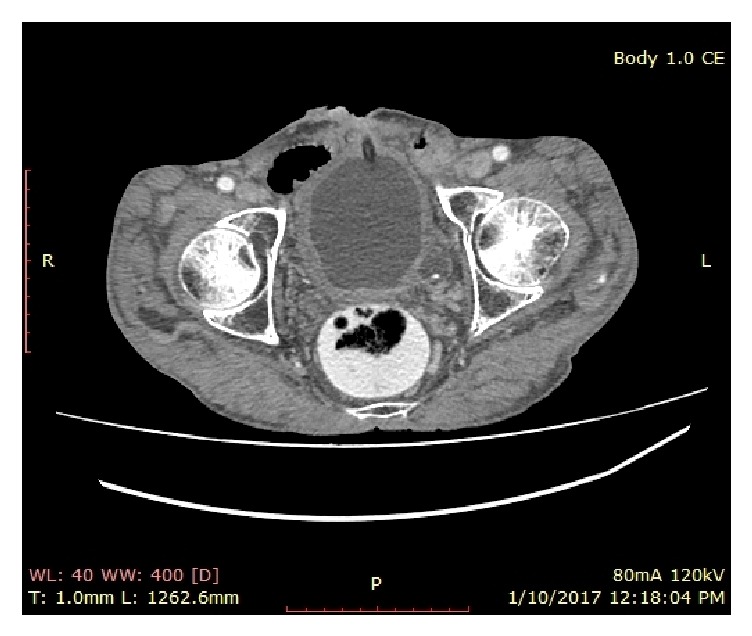
A contrast enhanced computed tomography scan image of suprapubic cystostomy site growth, transverse section.

**Figure 3:**
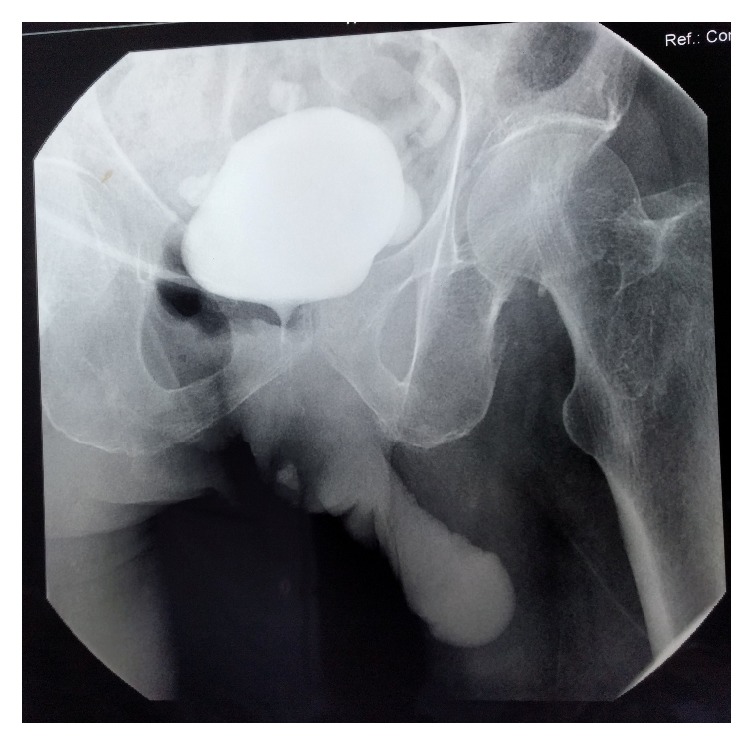
Cystogram performed through suprapubic cystostomy demonstrates obstruction beyond the prostatic urethra.

Physical examination revealed a fungating growth surrounding the suprapubic cystostomy site (Figure 1). Biopsy of the lesion showed squamous cell carcinoma. Contrast enhanced computed tomography scan of the abdomen and pelvis revealed a tumor around the cystostomy tract without involving the bladder and lymph nodes were negative (Figure 2). The upper tracts were normal and his renal function test was normal with serum creatinine of 90 *μ*mol/L. Flexible cystoscopy through the cystostomy revealed no evidence of growth in the bladder. Cystogram was done to assess the possibility of perineal urethrostomy to place the urinary diversion after resection (Figure 3). The cystogram showed the obstruction from the level of bladder neck and eliminated perineal urethrostomy as an option.

**Figure 4:**
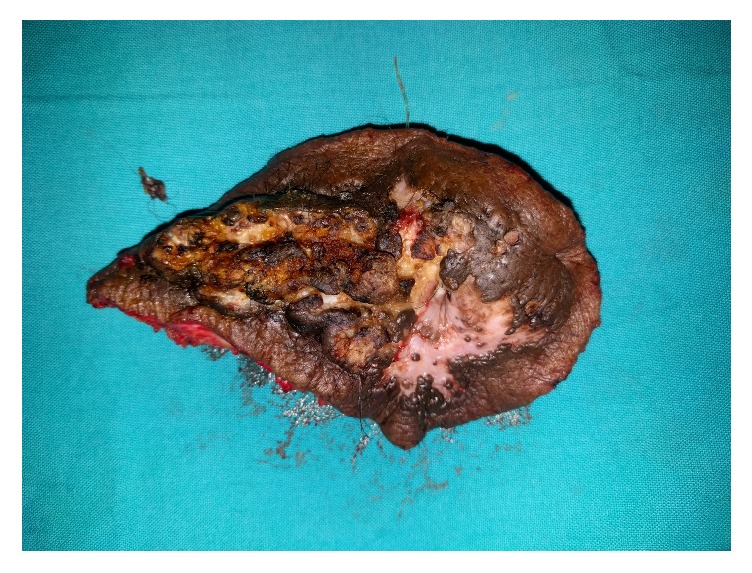
Wide local excision of the growth.

The surgery was performed as a combined procedure by both urology and plastic surgery team under general anesthesia. The growth was excised with a macroscopic margin of 1 cm from skin down to the rectus sheath and cuff of the bladder with the cystostomy tract also removed en bloc (Figure 4). Intraoperatively, we found that the tumor did not invade into the bladder.

**Figure 5:**
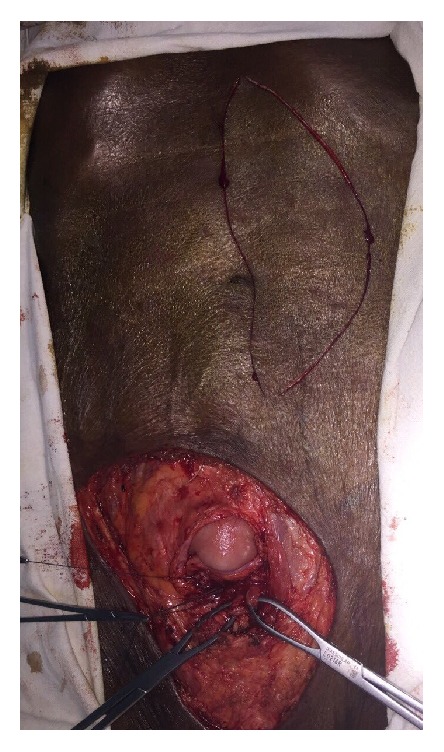
After the tumor resection, the skin incision lines to raise the flap are illustrated.

Full-thickness reconstruction was done with inferior epigastric artery based vertical rectus abdominis myocutaneous pedicled flap from the left side. As shown in Figure 5, skin incision was made vertically and the width of the skin paddle was decided by the defect in the excision site in a way to close the defect. The myocutaneous flap was harvested while preserving the posterior rectus sheath and the flap was delivered through the subcutaneous tunnel into the defect site without compromising the vasculature.

**Figure 6:**
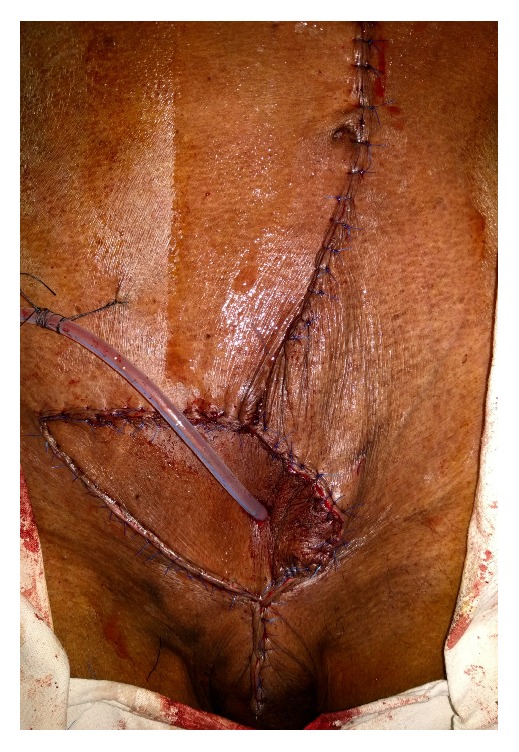
Photograph of completion of reconstruction, showing that the urinary catheter is exteriorized through the flap.

A silicon catheter was passed through the center of the flap while taking care not to damage the vascular supply to the flap (Figure 6). The catheter tip was placed into the bladder through the defect and the bladder defect was closed. The bladder wall was anchored to the under surface of the flap. The anterior rectus sheath of the flap was sutured to the muscular aponeurotic defect with size 2/0 polypropylene suture and then the deep dermal layers and skin were repaired accordingly. The laxity of the abdominal wall allowed the closure of the donor defect primarily with polyamide sutures.

**Figure 7:**
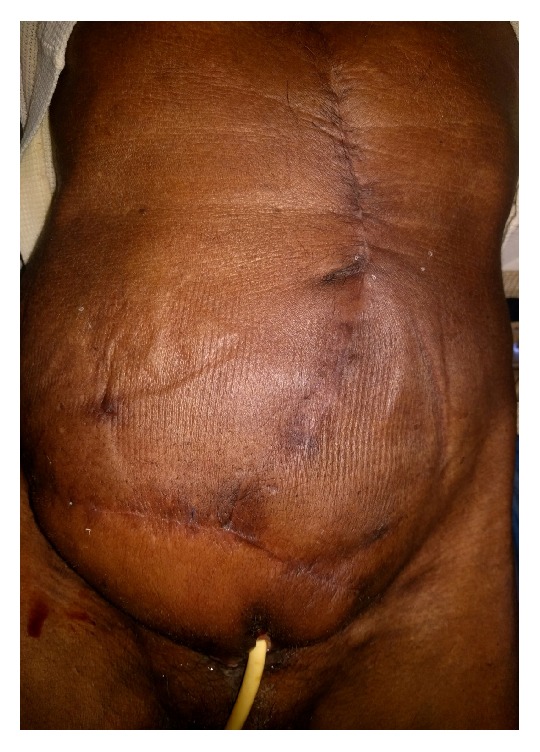
Healed wound at 6 months of follow-up.

Postoperatively, the patient developed mild urine leak through the inferior border of the flap, but it settled with conservative measures by day 10. Otherwise, the patient had uncomplicated recovery after surgery.  Figure 7 illustrates the healed surgical wound.

**Figure 8:**
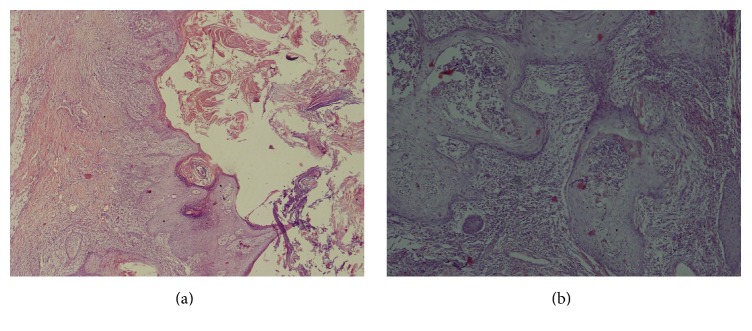
Well differentiated squamous cell carcinoma at low power (×40) (a) and high power (×100) (b).

Histopathology report revealed a well differentiated early invasive squamous cell cancer extending into the dermis (pT2), and the deep and radial resection margins were free of tumor (Figure 8).

The patient did not undergo any other additional treatment modalities. At the follow-up of six months, the patient was without any symptoms. The abdominal wall strength was satisfactory and there was no incisional hernia. We plan to follow up this patient three-monthly.

## 3. Discussion

Chronic suprapubic catheterization may rarely give rise to SCC, and in the literature, there are few reported cases. Chronic irritation and metaplasia are proposed to be the pathophysiology in a number of studies [11–13]. In 1993, Stroumbakis et al. reported the first patient with SCC on long-term suprapubic cystostomy following urethral stricture [2]. Since then, there have been a number of reported cases in the literature regarding suprapubic cystostomy site squamous cancer with or without bladder involvement, which are summarized in Table 1.

According to Table 1, the indication for suprapubic cystostomy is either neurogenic bladder drainage or urethral stricture. The median age at presentation was 61 years (range: 40–88, mean: 65). Seven out of 11 patients have been on suprapubic cystostomy for more than 20 years, even though the duration of suprapubic cystostomy is in the range of 9 months to 35 years [2–10]. Some patients having a past history of suprapubic cystostomy also developed SCC at the scar site [5, 9]. This reveals that the initial insult itself can predispose to a malignant change. Almost all the cases in the literature were advanced disease (T4) compared to the present case (T2). SCC at the suprapubic cystostomy site can present with or without bladder involvement. Including the present case, the patient without bladder involvement underwent limited resection in the form of partial cystectomy or wide local excision [2, 4]. Either surgery or radiotherapy or both were used as modalities of treatment in SCC. In the present case, considering the patient's age and sparing of the bladder from tumor invasion create the possibility of going for limited resection with placement of a urinary catheter through the flap. In most cases, treatment modalities were used as a palliative measure. But in the present case we opt for curative resection. With the support of the plastic surgery team, reconstruction was done and suprapubic cystostomy was taken through the flap which was not previously described in the literature. As the resection was done with negative margins and there was no evidence of metastasis, the multidisciplinary team's decision was to follow him up with active surveillance without any further adjuvant treatment.

## 4. Conclusion

It is prudent to look for changes around the suprapubic cystostomy site in a patient on long-term suprapubic catheterization or who had a suprapubic cystostomy in the past. It has to be carefully evaluated for malignancy. Even though SCC in the suprapubic cystostomy site is rare, early identification leads to a tumor-free resection margin at surgery and the ability to avoid extensive resection, such as cystectomy.

## Figures and Tables

**Table 1:** Reported cases of suprapubic cystostomy site SCC and details.

Reporting year	Study	Age	Indication for suprapubic cystostomy	Duration of suprapubic cystostomy	Bladder involvement	T stage	Modality of treatment	Survival
1993	Stroumbakis et al. [2]	80	Urethral stricture	5 years	No	T4	Radiation and excision	—
1995	Stokes III et al. [3]	50	—	25 years	Yes	T4	Surgery	8 months
1999	Schaafsma et al. [4]	80	—	5 years	No	T4	Wide excision and partial cystectomy	5 months
2000	Gupta et al. [5]	40	Urethral stricture (healed tract)	20 years	Yes	T4	Radical cystoprostatectomy and excision of mass with ileal conduit	>3 months
2011	Ito et al. [6]	58	Paraplegia	35 years	Not mentioned	T4	Radiation	>6 months
2013	Chung et al. [7]	56	Urethral stricture	9 years	Yes	T4	Radiation	6 months
2014	Massaro et al. [8]	55	Paraplegia	38 years	Yes	T4	Surgical resection and recurrence in 1 year end up in palliative care	Not available
2014	Massaro et al. [8]	85	Idiopathic urinary retention	9 months	Yes	T4	Surgical resection and palliative care	Not available
2015	Ranjan et al. [9]	68	Urethral stricture (multiple suprapubic cystostomy)	20 years (initial suprapubic cystostomy)	Yes	T4	Radiotherapy	4 months
2015	Zhang et al. [10]	61	Paraplegia	28 years	Yes	T4	Radiotherapy	>2 years
2017	Present study	88	Urethral stricture	25 years	No	T2	Wide local excision with bladder cuff and flap reconstruction	Survival at follow-up of 6 months
